# A nomogram for predicting overall survival in patients with Ewing sarcoma: a SEER-based study

**DOI:** 10.1186/s12891-020-03706-3

**Published:** 2020-11-12

**Authors:** Zhenggang Zhou, Jinyu Wang, Liming Fang, Jianlin Ma, Mingbo Guo

**Affiliations:** 1 Department of Spine Surgery, Qingdao Chengyang People’s Hospital, No.600 Changcheng Road, Chengyang District, Qingdao, Shandong Province 266109 People’s Republic of China; 2 Department of Orthopaedics, Weifang Sunshine Union Hospital, Weifang, Shandong 261000 People’s Republic of China

**Keywords:** Nomogram, SEER, Ewing sarcoma, Overall survival

## Abstract

**Background:**

Ewing sarcoma, the second most frequent bone tumor in children and adolescents, is often presented with localized disease or metastatic-related symptoms. In this study, we aim to construct and validate a nomogram for patients with Ewing sarcoma to predict the 3- and 5-year overall survival (OS) based on the Surveillance, Epidemiology, and End Results (SEER) database.

**Methods:**

Demographic and clinic pathological characteristics of patients with Ewing sarcoma diagnosed between 2010 and 2015 were extracted from SEER database. Univariate and multivariate Cox analyses were carried out to identify the independent characteristics. The independent factors were further included into the construction of a nomogram. Finally, c-index and calibration curves were used to validate the nomogram.

**Results:**

A total of 578 patients were enrolled into our analysis. The results of univariate Cox analysis showed that age, 7th AJCC stage, 7th AJCC T stage, 7th AJCC N stage, 7th AJCC M stage, metastatic status to lung, liver and bone were significant factors. Multivariate Cox analysis was performed and it confirmed age, N stage and bone metastasis as independent variables. Next, a nomogram was constructed using these independent variables in prediction to the 3- and 5-year OS. Furthermore, favorable results with c-indexes (0.757 in training set and 0.697 in validation set) and calibration curves closer to ideal curves indicated the accurate predictive ability of this nomogram.

**Conclusions:**

The individualized nomogram demonstrated a good ability in prognostic prediction for patients with Ewing sarcoma.

## Background

Ewing sarcoma, the second most frequent bone tumor in children and adolescents, is often presented with localized disease or metastatic-related symptoms [1]. There are several clinical parameters influencing the survival of patients with Ewing sarcoma. Age, tumor stage, tumor location, metastatic disease, chemotherapy and surgery have been found to have impacts on overall survival (OS) in patients with Ewing sarcoma [2–7]. The aim of this study is to integrate prognostic parameters into analysis and predict outcomes in patients with Ewing sarcoma.

As a statistical prognostic model, the nomogram represents a pattern of graph, in which variables are given marks, and therefore it easily assesses the probability of a certain event, in comparison with traditional evaluation standards [8]. In recent years, this model has been widely applied as the increased need of individualized medicine in a great variety of tumors [9–12]. Consequently, in the present study, we extracted data in patients with Ewing sarcoma from the Surveillance, Epidemiology, and End Results (SEER) database, to construct and validate a nomogram for predicting OS.

## Methods

### Study population

The SEER database provides demographic and clinical pathologic information of patients in the United States. Data in this study were further obtained from the SEER 18 Regs (1973–2015 varying). Patients who were included into the analysis would meet the following criteria: Ewing sarcoma cases (histological code 9260/3) diagnosed from 2010 to 2015, only one primary tumor in “bones and joints”. Cases diagnosed made by death certificate or autopsy were excluded from our analysis. The variables in our research included age, race, sex, 7th AJCC stage, 7th AJCC T stage, 7th AJCC N stage, 7th AJCC M stage, and metastatic status to lung, brain, liver and bone. Overall survival was defined as the period from diagnosis to death or time of the last follow-up.

### Nomogram construction

The patients were divided into a training set (*n* = 406) and a validation set (*n* = 172) by performing the package of caret (Classification and Regression Training) in R version 3.6.1. The nomogram construction was based on the analysis in the training set. To identify factors related to prognosis, univariate and multivariate Cox proportional hazards regression analysis were performed. The results of multivariate Cox regression analysis were further used to formulate the nomogram by performing rms package in R version 3.6.1.

### Nomogram validation

Two criteria, concordance index (c-index) and the calibration curve, were used to validate the nomogram model in both the training and the validation sets. C-index, a value range between 0 and 1, is to assess performance of the model. The larger c-index (> 0.70) is, the better performance the model has [10]. Calibration curves closer to ideal ones were thought to have the accurate predictive ability of this nomogram [11].

### Statistical analysis

All statistical analyses were performed in R 3.6.1 (http://www.Rproject.org). *P* < 0.05 was considered statistically significant. The nomogram construction was based on Cox proportional hazard regression models. Kaplan-Meier method was used to display OS curves by survival and survminer packages in R 3.6.1.

## Results

### Demographics and clinic pathological characteristics of the training and validation sets

**Fig. 1:**
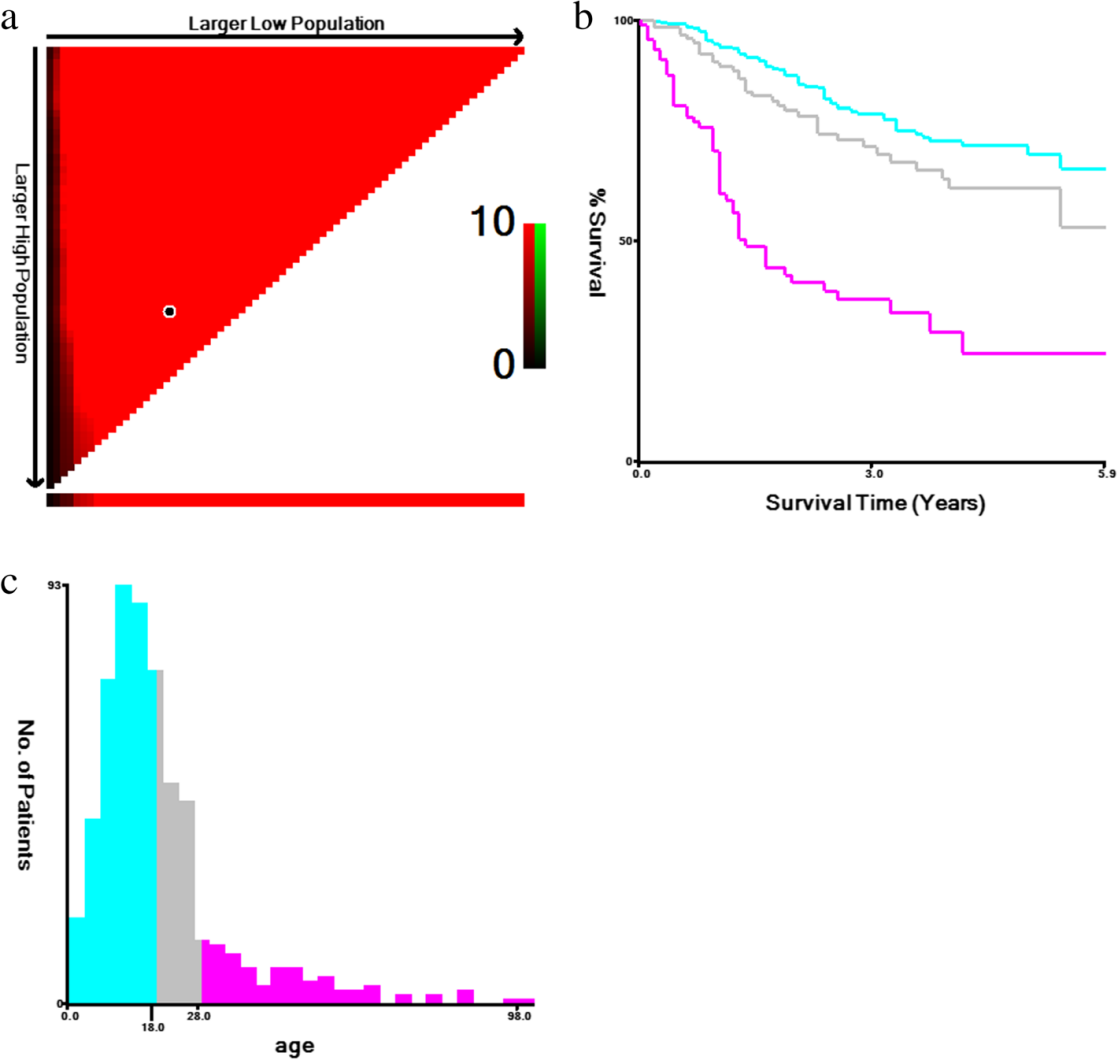
Identification of optimal cutoff values of age of diagnosis via X-tile analysis. **a** The vertical axis represents all possible high OS populations, with the size of the high OS population increasing from top to bottom. The horizontal axis represents all possible low OS populations, with the size of the low OS population increasing from left to right. Coloration of the plot represents the strength of the association at each division, ranging from low (dark, black) to high (red). **b** Kaplan-Meier survival curves of three age groups. **c** The histogram of three age groups OS, overall survival.

A total of 578 patients with Ewing sarcoma from the SEER database diagnosed from 2010 to 2015 were incorporated into the present study. The value of age was transformed into three categorical variables: ≤18, 19–27 and ≥ 28 years by performing X-tile (Fig. 1). As Table 1 showed, the demographic and clinicopathological characteristics of these two sets were similar.

**Table 1:** Demographics and clinicopathological characteristics of patients with Ewing sarcoma

Characteristics	Training set(*n* = 406)	Percent (%)	Validation set(*n* = 172)	Percent (%)
	Number of patients		Number of patients	
Age (years)				
≤ 18	248	61.1	107	62.2
19–27	89	21.9	38	22.1
≥ 28	69	17.0	27	15.7
Sex				
Male	250	61.6	104	60.5
Female	156	38.4	68	39.5
Race				
White	344	84.7	155	90.1
Black	18	4.5	6	3.5
Others/unknown	44	10.8	11	6.4
stage				
II	212	52.1	88	51.2
III	6	1.5	1	0.6
IV	141	34.7	57	33.1
Others/unknown	47	11.7	26	15.1
T stage				
T0-T1	165	40.6	59	34.3
T2	143	35.2	67	39.0
T3	21	5.2	8	4.6
Tx	77	19.0	38	22.1
N stage				
N0	345	85.0	147	85.5
N1	28	6.9	12	7.0
Nx	33	8.1	13	7.5
M stage				
M0	274	67.5	117	68.0
M1	132	32.5	55	32.0
Bone metastasis				
Yes	56	13.8	26	15.1
No/unknown	350	86.2	146	84.9
Brain metastasis				
Yes	4	1.0	2	1.2
No/unknown	402	99.0	170	98.8
Liver metastasis				
Yes	4	1.0	2	1.2
No/unknown	402	99.0	170	98.8
Lung metastasis				
Yes	71	17.5	37	21.5
No/unknown	335	82.5	135	78.5

### Identification of prognostic factors in the training set

**Fig. 2:**
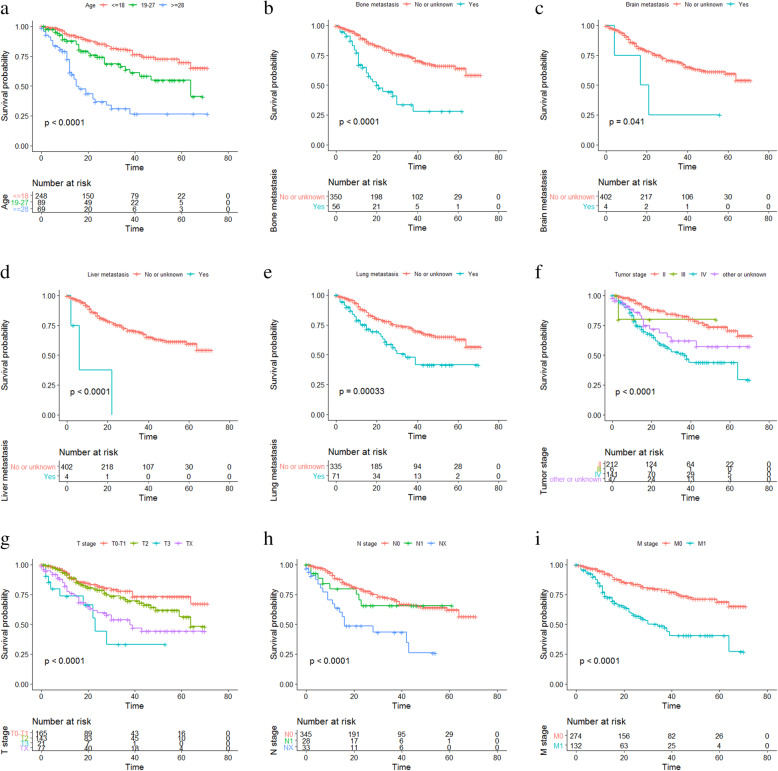
Kaplan-Meier survival curves of OS. Kaplan-Meier survival curves based on **a** age, **b** bone metastasis, **c** brain metastasis, **d** liver metastasis, **e** lung metastasis, **f** tumor stage, **g** T stage, **h** N stage, **i** M stage. OS, overall survival

Univariate Cox analysis was carried out to work out the effect of demographics and clinic pathological characteristics on survival outcomes. As shown in Table 2, age, 7th AJCC stage, T stage, M stage, N stage, and the metastatic status to the liver, lung and bone were risk factors in patients with Ewing sarcoma. Multivariate Cox analysis was further performed and suggested that age, N stage and bone metastasis were independent prognostic factors for OS. In addition, Kaplan-Meier curve analysis was used to verify the prognostic abilities of these factors (Fig. 2a-j), indicating that longer OS was related to younger age (*p* < 0.0001), lower tumor stage (*p* < 0.0001), lower M stage (*p* < 0.0001), lower T stage (*p* < 0.0001), lower N stage (*p* < 0.0001), no metastasis to the bone and liver (*P* < 0.0001), no metastasis to the brain (*P* = 0.041), no metastasis to the lung (*P* = 0.00033). Gender (*P* = 0.9) and race (*P* = 0.26) had no significant impact on OS (Additional file 1).

**Table 2:** Hazard ratio of overall survival for patients with Ewing sarcoma based on Cox regression

Characteristics	Univariate analysis	95% CI	*P* value	Multivariable analysis	95% CI	*P* value
	Hazard ratio			Hazard ratio		
Age (years)						
≤ 18	Reference			Reference		
19–27	1.928	1.200–3.097	0.007	1.778	1.092–2.895	0.021
≥ 28	5.324	3.434–8.254	<0.001	5.145	3.231–8.192	<0.001
Sex						
Male	Reference			Not included		
Female	1.026	0.700–1.502	0.897			
Race						
White	Reference			Not included		
Black	1.828	0.887–3.767	0.102			
Others/unknown	1.075	0.560–2.066	0.827			
stage						
II	Reference			Reference		
III	2.022	0.276–14.793	0.488	2.545	0.289–28.446	0.400
IV	3.266	2.142–4.977	<0.001	1.100	0.117–10.312	0.933
Others/unknown	2.144	1.168–3.937	0.014	1.195	0.484–2.945	0.699
T stage						
T0-T1	Reference			Reference		
T2	1.384	0.857–2.234	0.184	1.284	0.769–2.144	0.340
T3	3.905	1.845–8.264	<0.001	1.367	0.558–3.347	0.494
Tx	2.558	1.565–4.181	<0.001	1.453	0.731–2.888	0.286
N stage						
N0	Reference			Reference		
N1	1.335	0.646–2.758	0.436	0.686	0.258–1.825	0.450
Nx	3.188	1.912–5.312	<0.001	3.392	1.848–6.224	<0.001
M stage						
M0	Reference			Reference		
M1	2.992	2.055–4.356	<0.001	1.981	0.205–19.147	0.555
Bone metastasis						
No/unknown	Reference			Reference		
Yes	3.476	2.271–5.320	<0.001	2.309	1.315–4.056	0.004
Brain metastasis						
No/unknown	Reference			Not included		
Yes	3.087	0.979–9.735	0.054			
Liver metastasis						
No/unknown	Reference			Reference		
Yes	8.918	2.801–28.390	<0.001	2.861	0.651–12.564	0.164
Lung metastasis						
No/unknown	Reference			Reference		
Yes	2.121	1.393–3.227	<0.001	1.226	0.699–2.148	0.477

### Construction of the nomogram in the training set

**Fig. 3:**
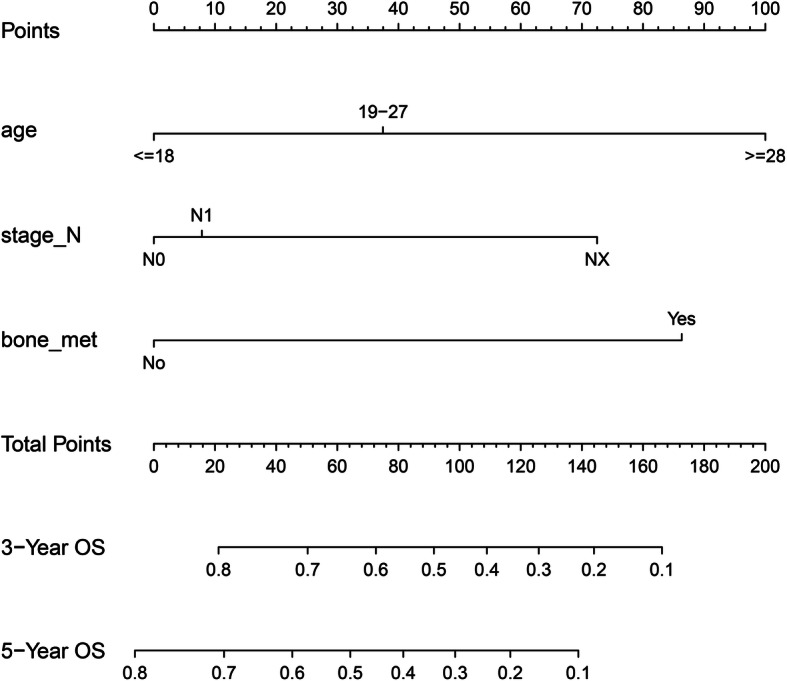
Nomogram for the prediction of 3- and 5-year OS in patients with Ewing sarcoma. OS, overall survival

To explore a quantitative approach to predicting 3- and 5-year OS, a nomogram that included all the clinic pathological independent risk factors was formulated (Fig. 3). The scores of the items displayed in the nomogram should be added up. As it showed in Fig. 3, age contributed most to prognosis, followed by bone metastasis and N stage.

### Validation of predictive accuracy of the nomogram in the training and validation sets

**Fig. 4:**
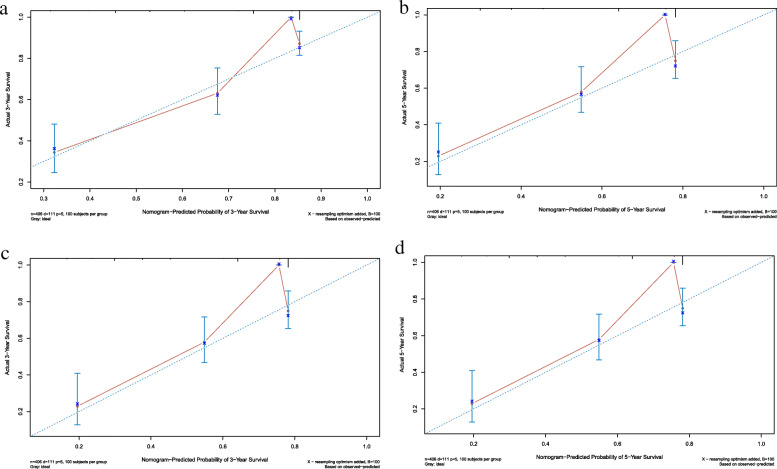
Calibration curves of the nomogram predicting 3- and 5-year OS in patients with Ewing sarcoma. **a** Calibration curve of 3-year OS in the training cohort. **b** Calibration curve of 5-year OS in the training cohort. **c** Calibration curve of 3-year OS in the validation cohort. **d** Calibration curve of 5-year OS in the validation cohort. OS, overall survival

To validate the predictive accuracy of the nomogram, c-index and calibration curves were used to evaluate this model. C-indexes were observed in both the training (0.757) and validation sets (0.697), which suggested the good accuracy of this model. Next, the packages of rms, foreign and survival were performed in R 3.6.1, and high agreements between ideal curves and calibration curves were observed in both training and validation sets (Fig. 4a-d). These results revealed a good discrimination ability of the nomogram model.

## Discussion

In this study, we built an individualized nomogram, which integrated routinely available information such as age, N stage, and metastasis to bone, to predict OS in a large cohort of patients with Ewing sarcoma. C-indexes and calibration curves were used in the validation.

The results of Kaplan-Meier curve showed that gender had no significant impact on OS, which was consistent with that of S.E. Bosma, Friedman Danielle Novetsky and Ren Yingqing et al. [3, 4, 7], who found that female patients with Ewing sarcoma had similar OS to male patients. In the process of developing the nomogram, we found that age played a pivotal role in total points. Those aged over 28 had a high risk and a shorter OS (19–27 years: hazard ratio (HR) =1.928, 95% confidence interval (CI) = 1.200–3.097; ≥28 years: HR = 5.324, 95% CI = 3.434–8.254). This finding was consistent with the results of other studies [3–6], except S.E. Bosma et al., who indicated by a system review that the level of evidence for an association with OS for age was inconclusive.

In childhood and adolescence, tumor metastasizes to the liver, bone and lung at an early stage [13, 14]. Despite timely treatment, patients suffered from metastasis usually have a poor OS [15–17]. Accordingly, the multivariate Cox analysis results revealed that metastasis to the bone was another independent factor for OS, and patients with bone metastasis in our analysis lived shorter than those without metastasis (bone metastasis: HR = 3.476, 95% CI = 2.271–5.320). In addition, as we constructed the nomogram, N stage (AJCC, 7th ed.) was also taken into account. The applications of nomogram models in several tumors were found to have a better prognostic performance than the staging systems alone [10, 11]. In intrahepatic cholangiocarcinoma patients who underwent partial hepatectomy, Wang et al. [10] included both laboratory indices and demographic data in construction of nomogram, finding that this nomogram was more accurate in predicting OS than different staging systems. Wang et al. [11] combined staging system with demographic information of patients and then developed a nomogram, leading to a similar conclusion. Taken together, the nomogram combining demographics with staging system predicted OS in a more accurate way.

This study had some limitations. First, although c-indexes and calibration curves had been applied to validate the nomogram, the present research lacked external validation. More work should be done to strengthen the validity of the model. Second, the data of treatment were not collected, so that the predictive value of OS was not absolutely precise due to the fact that survival is affected partly by the treatment [18]. However, not including surgery, chemotherapy and radiotherapy in the nomogram could make this model more applicable to patients initially presenting to clinic who are waiting for evaluation from oncologists. Third, this model was constructed based on a retrospective cohort, which means that the inherent biases were unavoidable. Thus further prospective researchers are required for validation.

## Conclusions

The nomogram demonstrated a good ability in prognostic prediction for patients with Ewing sarcoma.

## Supplementary information

**Additional file 1.**

## Data Availability

The raw data in our study are in the SEER database and a registered account is required for requesting data. The datasets supporting the conclusions of this article are included within the article and its additional files.

## Abbreviations

OS: Overall survival
SEER: Surveillance, Epidemiology, and End Results
c-index: Concordance index
HR: Hazard ratio
CI: Confidence interval
