# Supplementary figures and images for: A nomogram for predicting overall survival in patients with Ewing sarcoma: a SEER-based study
Source: BMC Musculoskelet Disord. 2020 Nov 12;21:737. doi: 10.1186/s12891-020-03706-3 (PMC7661249; doi:10.1186/s12891-020-03706-3)

a

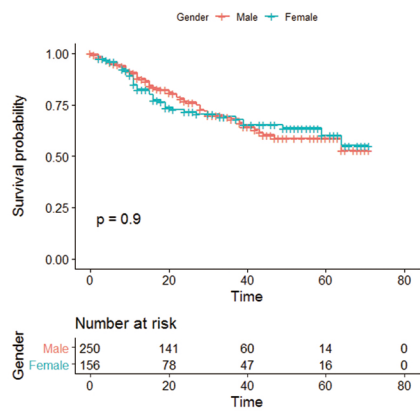

b

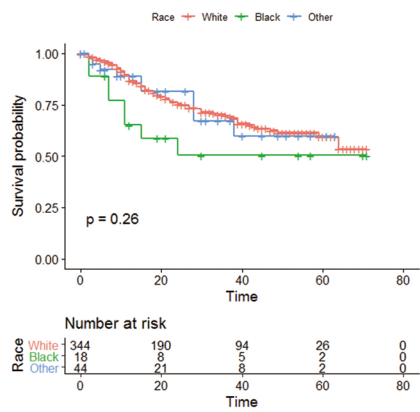

Supplement: Supplementary file 1 — Additional file 1. [file 12891_2020_3706_MOESM1_ESM.pdf]
